# The Evaluation of YouTube™ Videos Pertaining to Intraoperative Anaesthesia Awareness: A Reliability and Quality Analysis

**DOI:** 10.7759/cureus.35887

**Published:** 2023-03-08

**Authors:** Fatma F Kartufan, Erkan Bayram

**Affiliations:** 1 Department of Anesthesia and Reanimation, Istinye University Medical Park Gaziosmanpasa Hospital, Istanbul, TUR

**Keywords:** reliability, youtube™, discern, gqs, anaesthesia awareness

## Abstract

Aim

The objective of this study was to evaluate the reliability and quality of YouTube™ (Google, Inc., Mountain View, CA) videos pertaining to anaesthesia awareness.

Methods

We evaluated the most commonly viewed 100 videos pertaining to anaesthesia awareness. The YouTube™ videos’ image type, qualification of the uploaders, video content, video length in minutes, upload time, time since upload, total view count, daily view count and comment and like counts were recorded. The quality of the YouTube™ videos was evaluated using the Global Quality Scale (GQS), and the reliability was determined using the modified DISCERN scale.

Results

Of all videos, 34 (34%) were uploaded directly by physicians, 16 (16%) by patients, 14 (14%) by health channels, 13 (13%) by TV shows and 23 (23%) by others. The mean video length was 11.48±11.96 minutes. The average DISCERN score was 4.47±0.58 in the professional and 3.28±0.65 in the non-professional video group (p<0.001). The mean GQS score was 4.47±0.52 in the professional and 3.35±0.67 in the non-professional video group (p<0.001).

Conclusion

The results of this study indicate that a significant portion of the YouTube™ videos pertaining to anaesthesia awareness were uploaded directly by physicians or by health channels. Physicians and professional health institutions should be promoted to provide accurate and more reliable videos to direct patients to the right solutions for their problems. YouTube™ videos should be subjected to supervision before they can be publicly viewed.

## Introduction

Awareness is becoming conscious during some part of the operation. Anaesthesia awareness is the postoperative recall of events experienced under general anaesthesia. This phenomenon refers to the situation when we cannot assume that anaesthesia is not of adequate depth based on usual clinical signs including blood pressure increase, heart rate frequency, muscular contractions and lacrimation [1]. Anaesthesia awareness is one of the common concerns expressed by patients who are about to undergo anaesthesia [2]. Many anaesthesiologists are reporting an increase in the number of patients raising questions about intraoperative awareness. One or two in every 1,000 patients who receive general anaesthesia experience anaesthesia awareness [3]. As in many other health-related situations, patients who are about to undergo surgery seek information from online sources regarding their concerns about anaesthesia awareness instead of getting information from health professionals.

In the twenty-first century, people are increasingly using the internet to obtain health-related information [4]. The internet is becoming a significant source for patients and their relatives [5]. Patients obtain information on the internet before seeking help from a professional and as a second opinion [6]. Internet users usually have high confidence in information found online [7]; however, many websites contain incorrect or misleading information [8]. It has been reported that about 70% of internet users first search online sources when they have a health-related problem [9].

With more than 2.6 billion monthly active users, YouTube™ (Google, Inc., Mountain View, CA) is the largest online video-sharing platform and is the second most-used website following Google (Google, Inc., Mountain View, CA) [10]. Every minute, 500 videos are uploaded to YouTube™, which is the primary source of video for 78% of internet users [10]. YouTube™ now includes a growing health-related video archive for medical students, doctors, patients and relatives [7]. YouTube™^ ^has several advantages as a source of health-related information. YouTube™ videos are publicly available for views and downloads. However, there are concerns over the reliability and quality of information on this platform since the uploaded videos do not pass a peer review or quality control [11]. Some health-related videos may contain incorrect, out of date and misleading information that can be rapidly disseminated with negative consequences [12].

In searching the literature, we could not find studies evaluating YouTube™ videos on anaesthesia awareness. Therefore, the objective of this study was to evaluate the reliability and quality of YouTube™ videos pertaining to anaesthesia awareness.

## Materials and methods

**Figure 1:**
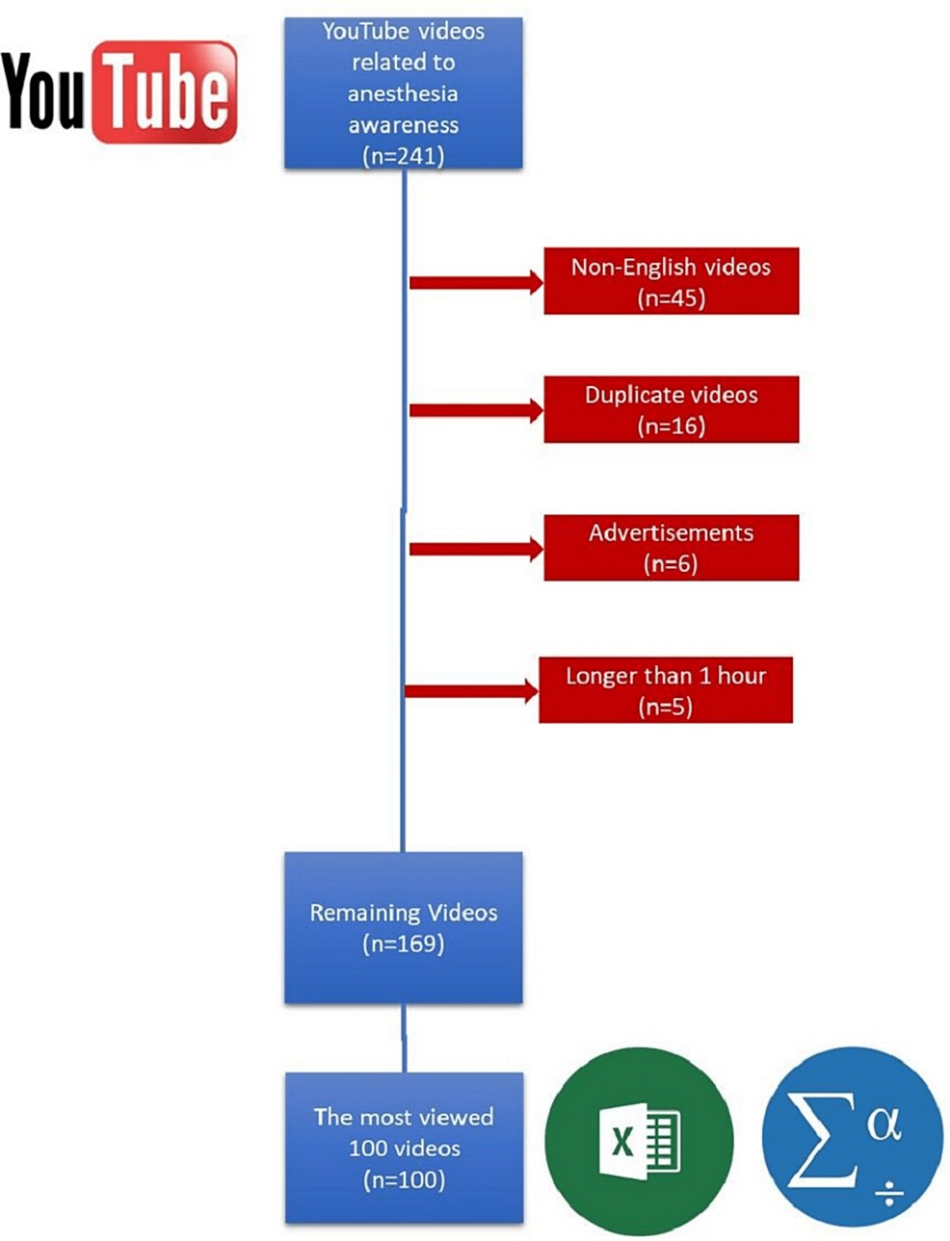
Inclusion of YouTube™ videos related to anaesthesia awareness

This cross-sectional study was conducted by two anaesthesiologists from October 10th to 14th, by evaluating YouTube™ videos related to anaesthesia awareness. No ethical approval or informed consent was needed as the study did not involve animal or human subjects. The search terms were determined by the consensus of the two researchers as ‘anaesthesia awareness’ and ‘anaesthetic awareness’. The videos were sorted according to the ‘view’ option from the filter on YouTube™. Accordingly, 241 relevant videos were obtained. From the results, advertisements, non-English videos, duplicate ones and those longer than one hour were excluded from the study. The inclusion flowchart is shown in Figure 1.

Although there are several studies investigating all videos on the relevant topic, the commonly used method is to include a fixed size. Therefore, we evaluated the 100 most-viewed videos. The links for these 100 videos were entered into Microsoft Excel (Microsoft® Corp., Redmond, WA). The evaluation of the videos was performed by two experienced anaesthesiologists (eight and 10 years) at the same time but in different locations to prevent potential bias.

Data collection

The most commonly viewed YouTube™^ ^videos’ image type (real or animation), qualification of the uploaders (physicians, health channels, TV shows, patients or others), video content (general information, patient experience or prevention), video length in minutes, upload time, time since upload, total view count, daily view count and comment and like counts were recorded. The quality of the YouTube™ videos was evaluated using the Global Quality Scale (GQS), and the reliability was determined using the modified DISCERN scale. We grouped the YouTube™ videos by those uploaded by professionals and those uploaded by non-professionals. Although the content of some videos uploaded by health channels had been narrated by physicians, most of them were narrated by other persons or included advertisements; thus, we included these videos in the non-professional group.

Modified DISCERN scale

The modified DISCERN scoring is a method for the evaluation of the reliability of consumers’ healthcare information on various treatment options. A five-point modified DISCERN score was used to evaluate the reliability of the videos. Each question in this tool is scored between 1 and 5 with higher scores indicating better reliability. The reliability of a video was considered good for a DISCERN score of >3 points, moderate for a score of 3 points and poor for a score of <3 points [13-15].

Global Quality Scale (GQS)

The GQS, which was first developed by Bernard et al., was used to evaluate the quality of the videos [16]. The GQS has five items that inquire about the ease of use of the information and flow and quality of the examined videos. It is a five-point Likert scale. The GQS score of a video’s content is between 1 (very poor) and 5 (excellent).

Table 1 shows the questions included in the modified DISCERN scale and GQS.

**Table 1:** Modified DISCERN scale and GQS GQS: Global Quality Scale

DISCERN	Score	GQS
Is the video clear, concise and understandable?	1	Poor quality, poor flow of the site and most information missing: not at all useful for patients
Are reliable sources of information used?	2	Generally poor quality and poor flow and some information listed but many important topics missing: of very limited use to patients
Is the information presented balanced and unbiased?	3	Moderate quality, suboptimal flow and some important information adequately discussed but others poorly discussed: somewhat useful for patients
Are additional sources of information listed for patient reference?	4	Good quality and generally good flow and most of the relevant information listed but some topics not covered: useful for patients
Are areas of uncertainty/controversy mentioned?	5	Excellent quality and excellent flow: very useful for patients

Statistical analysis

The statistical analysis of the data obtained in this study was carried out utilizing the Statistical Package for Social Sciences (SPSS) version 25.0 (IBM SPSS Statistics, Armonk, NY) package software. The normality of the variables was studied with the Kolmogorov-Smirnov test. The comparison of continuous variables was made using the Mann-Whitney test or independent t-test according to the normality, while categorical variables were compared with the chi-square test. Continuous variables were expressed as mean±standard deviation and categorical variables as frequency (n, %). Pearson’s correlation analysis was used to determine the agreement between the two anaesthesiologists. A p-value of <0.05 was considered statistically significant.

## Results

**Figure 2:**
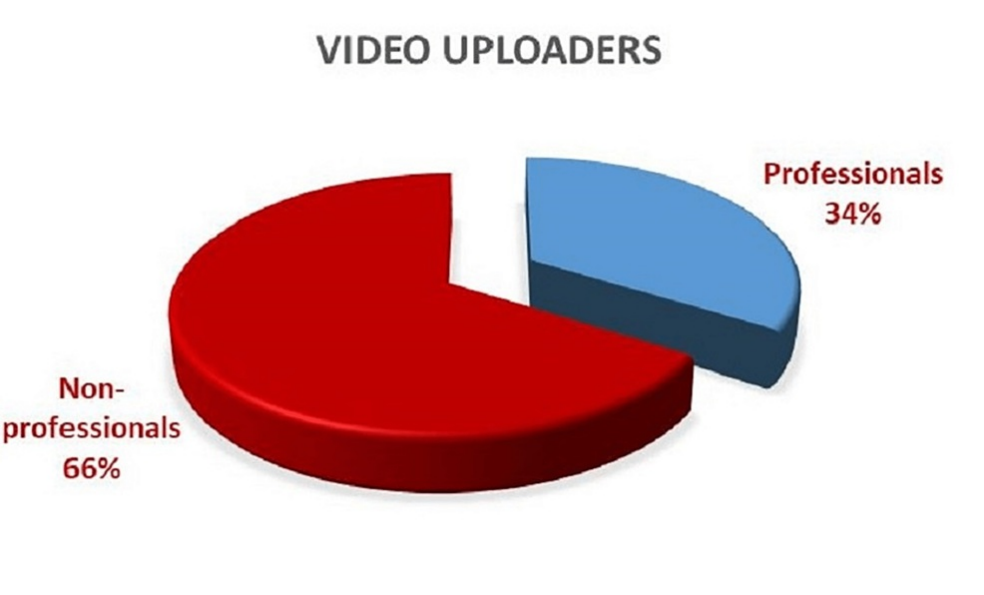
Distribution of the uploaders

A total of 100 most-viewed YouTube™ videos related to anaesthesia awareness were included in this study. The video image was real in 91 (91%) videos and animated in nine (9%) videos. Thirty-four videos were uploaded directly by medical physicians and 66 videos by non-professionals. Of all videos, 34 (34%) were uploaded directly by physicians, 16 (16%) by patients, 14 (14%) by health channels, 13 (13%) by TV shows and 23 (23%) by others. Figure 2 shows the distribution of YouTube™ videos by uploaders.

The total video length was 19.13 hours. The mean video length was 11.48±11.96 minutes. The mean video length was 15.63±17.19 minutes in the videos uploaded by professionals and 9.34±7.36 minutes in the videos provided by non-professionals. The average video length was significantly longer in video contents uploaded by professionals (p=0.012).

**Figure 3:**
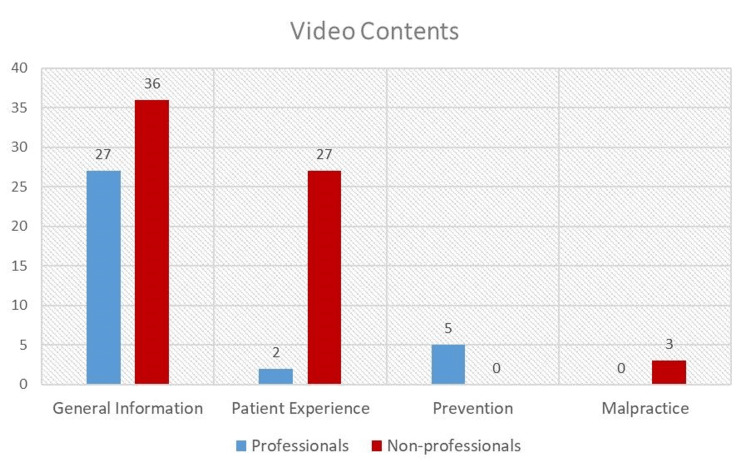
Distribution of video content by uploaders

The YouTube™ videos examined included general information in 63 (63%) videos, patient experience in 29 (29%) videos, prevention in five (5%) videos and malpractice in three (3%) videos. The distribution of video content by uploaders is given in Figure 3.

The mean daily view of the videos was 365.01±1217.09, the mean comment count was 1768.91±6722.94 and the mean like count was 10717.58±48973.48. The basic characteristics of the videos are given in Table 2.

**Table 2:** Basic features of the videos SD: standard deviation

Feature	Daily views		Comments		Likes	
	Mean	±SD	Mean	±SD	Mean	±SD
Image type						
Real (n=91)	383.00	1279.30	1820.49	7034.94	10775.24	50917.28
Animation (n=9)	203.16	299.03	1227.33	791.39	10025.71	10561.02
Uploaders						
Physicians (n=34)	60.19	220.84	166.46	635.24	794.34	3317.56
Health channels (n=14)	132.28	287.02	211.90	455.15	1119.07	2889.55
TV shows (n=13)	3146.72	3432.35	7022.89	14742.66	43639.17	115107.59
Patients (n=16)	371.83	864.78	3375.62	8816.84	19520.44	57334.07
Others (n=23)	114.93	241.72	680.92	788.00	5030.00	8416.44
Video content						
General information (n=63)	147.30	379.74	310.96	656.95	2367.69	5797.30
Patient experience (n=29)	873.27	2044.39	5386.85	11887.93	31029.07	87282.14
Prevention (n=5)	1.17	0.99	4.67	5.51	45.75	49.54
Malpractice (n=3)	0.79	0.14	0.00	0.00	3.00	1.41
Professionals versus non-professionals						
Physicians (n=34)	60.19	220.84	166.46	635.24	794.34	3317.56
Non-physicians (n=66)	545.14	1503.08	2623.56	8215.40	15359.10	58863.08

A comparison of the basic features of the videos uploaded by professionals and non-professionals is presented in Table 3.

**Table 3:** Comparison of basic characteristics between professionals and non-professionals

Feature	Professionals	Non-professionals	P-value
Daily views	60.19±220.84	545.14±1503.08	0.108
Comment count	166.46±635.24	2623.56±8215.40	0.150
Like count	794.34±3317.56	15359.10±58863.08	0.188

Accordingly, there was no statistically significant difference between the video contents provided by professionals and non-professionals in terms of daily views, comment count and like count (for all, p>0.05).

The mean DISCERN score was 3.69±0.84, and the mean GQS score was 3.74±0.82 in all videos included in the study. The average DISCERN score was 4.47±0.58 in the professional and 3.28±0.65 in the non-professional video group (p<0.001). The mean GQS score was 4.47±0.52 in the professional and 3.35±0.67 in the non-professional video group (p<0.001). The distribution of the modified DISCERN and GQS scores according to the contents of the viewed videos is presented in Table 4.

**Table 4:** DISCERN and GQS scores according to the video contents GQS, Global Quality Scale; SD, standard deviation

Content	DISCERN		GQS	
	Mean	±SD	Mean	±SD
General information (n=63)	3.84	0.86	3.87	0.84
Patient experience (n=29)	3.22	0.84	3.31	0.82
Prevention (n=5)	4.6	0.78	4.6	0.77
Malpractice	3.33	0.88	3.5	0.87

According to the DISCERN scale, 14 (14%) of the YouTube™ videos related to anaesthesia awareness were of poor reliability, 17 (17%) were of moderate reliability and 69 (69%) were of good reliability. The distribution of video reliability by uploaders is given in Table 5.

**Table 5:** Videos’ reliability according to DISCERN scale by uploaders

	Low	Moderate	Good	Total
n (%)				
Physicians	1 (2.94)	0 (0)	33 (97.06)	34 (100)
Health channels	0 (0)	3 (21.43)	11 (78.57)	14 (100)
TV shows	1 (7.69)	5 (38.46)	7 (53.85)	13 (100)
Patients	4 (25.00)	6 (37.5)	6 (37.5)	16 (100)
Others	8 (34.78)	3 (13.04)	12 (52.18)	23 (100)

According to the GQS, 10 (10%) of the YouTube™ videos pertaining to anaesthesia awareness were of poor quality, 21 (21%) were of moderate quality and 69 (69%) were of good quality. The distribution of video reliability by uploaders is given in Table 6.

**Table 6:** Videos’ quality according to GQS by uploaders GQS: Global Quality Scale

	Low	Moderate	Good	Total
n (%)				
Physicians	0 (0)	1 (2.94)	33 (97.06)	34 (100)
Health channels	0 (0)	3 (21.43)	11 (78.57)	14 (100)
TV shows	1 (7.69)	5 (38.46)	7 (53.85)	13 (100)
Patients	1 (6.25)	9 (56.25)	6 (37.50)	16 (100)
Others	8 (34.78)	3 (13.04)	12 (52.18)	23 (100)

Pearson’s correlation analysis was performed to determine the agreement between the researchers as shown in Table 7.

**Table 7:** Agreement between the two anaesthesiologists GQS, Global Quality Scale; SD, standard deviation

	Mean±SD	r	p
DISCERN 1	3.65±0.87	0.759	p<0.001
DISCERN 2	3.72±0.92		
GQS 1	3.70±0.85	0.847	p<0.001
GQS 2	3.77±0.86		

Accordingly, there was excellent agreement between the researchers.

## Discussion

In this study, we found that a significant portion of the YouTube™ videos pertaining to intraoperative anaesthesia awareness were uploaded directly by physicians or by health channels. Sixty-nine percent of the videos were of good quality.

In recent years, patients and their relatives are increasingly seeking remedies and solutions to their health-related problems using the internet. They share their experiences and even buy treatment via the internet. It has been stated that the internet is the third most trustworthy source of healthcare information following physicians and healthcare institutions [17]. In addition to providing a platform for health-related information, the internet has created opportunities for open discussion about health-related topics. However, this opportunity brings with it some threats, such as misleading information due to its non-audited structure, especially since YouTube™ is a video-sharing platform where everyone can upload videos that can reach millions of people free of charge. The rise in published studies on the quality and reliability of YouTube™ video content is proof of this [18]. On the other hand, on January 26, 2021, a letter from a YouTube™ CEO declared that more than 500000 videos containing misinformation related to COVID-19 were removed from the platform according to their new health policies [19].

To the best of our knowledge, this is the first study to evaluate the reliability and quality of YouTube™ videos regarding anaesthesia awareness. In our study, 34 videos were uploaded directly by physicians, 16 by patients, 14 by health channels, 13 by TV shows and 23 by others. In a study by Kocyigit et al. investigating YouTube™ videos on pulmonary rehabilitation during COVID-19, 38 videos were uploaded by healthcare professionals, followed by health channels, universities, organisations, patients and independent users [12]. In another study by Akyol and Karahan investigating YouTube™ videos on sarcopenia, 34% of the videos were uploaded by healthcare professionals [20]. The rate of YouTube™ videos uploaded by healthcare professionals was similar among the studies.

The total video length was 19.13 hours, and the mean video length was 11.48±11.96 minutes. In a study by Baran and Baran investigating YouTube™ videos as an information source about urinary incontinence, the total video duration was 12.60 hours [21]. In another study by Sledzinska et al. investigating YouTube™ videos on meningioma treatment, the mean video length was 11.7±17.18 minutes [22]. In a study by Bahar-Ozdemir et al. investigating YouTube™ videos on cancer rehabilitation, the mean video length was 18.76±31.47 minutes [23]. In this context, our video length is consistent with those reported in the literature, although the video length may vary according to the topic of interest.

No significant difference was found between the videos uploaded by professionals and non-professionals in terms of daily views, comments and like counts, suggesting that patients tend to seek information through all YouTube™ videos without distinguishing the videos uploaded by healthcare professionals.

In the present study, we found that the DISCERN and GQS scores of the videos uploaded by healthcare professionals were statistically significant than those uploaded by non-professional persons (both p<0.001). Similarly, in a study by Turhan and Ünsal evaluating the quality of YouTube™ videos on haemorrhoidal disease, the DISCERN and GQS scores of the videos uploaded by healthcare professionals were statistically significantly higher than those uploaded by non-professional persons (both p<0.001) [24]. In another study by Tosun and Tosun [25], YouTube™ videos on blood pressure measurement were evaluated. As a result, the DISCERN and GQS scores were significantly higher in the videos uploaded by healthcare personnel compared to the others (p=0.024 and p<0.001, respectively). In an interesting study examining the quality of YouTube™ videos recommending exercises for the COVID-19 lockdown, the DISCERN scores were significantly higher in the videos uploaded by healthcare professionals [26].

In our study, the reliability of the YouTube™ videos was poor in 14%, moderate in 17% and good in 69%. The quality of the YouTube™ videos was poor in 10%, moderate in 21% and good in 69%. In a study by Kocyigit et al., quality was low in 34.92%, intermediate in 30.16% and high in 34.92% of the videos [12].

In the present study, an excellent agreement was observed between the two anaesthesiologists in terms of the modified DISCERN and GQS scores.

Study limitations

This study has several limitations. First, only the most commonly viewed 100 videos were analysed. Second, we evaluated only YouTube™^ ^videos that makes the generalisation of the results difficult. Finally, since YouTube™ has a dynamic nature, content, views, comments and likes change constantly, and we made a snapshot evaluation. However, this is the first study evaluating the reliability and quality of YouTube™ videos on anaesthesia awareness as a strength aspect.

## Conclusions

The results of this study indicate that a significant portion of the YouTube™ videos pertaining to anaesthesia awareness were uploaded directly by physicians or by health channels. The information provided by professionals was of higher quality than by non-professionals. Nevertheless, there are many misleading videos on this topic. Physicians and professional health institutions should be promoted to provide accurate and more reliable videos to direct patients to the right solutions for their problems. YouTube™ videos should be subjected to supervision before they can be publicly viewed.
